# The role of institutions on the effectiveness of malaria treatment in the Ghanaian health sector

**DOI:** 10.1186/s12913-015-0802-7

**Published:** 2015-04-19

**Authors:** Eugenia Amporfu, Justice Nonvignon

**Affiliations:** Kwame Nkrumah University of Science and Technology, Kumasi, Ghana; School of Public Health, University of Ghana, Legon, Ghana

**Keywords:** Institutions, Decentralization, Effectiveness of treatment, Health outcome

## Abstract

**Background:**

The Ghanaian health sector has undertaken several policies to help improve the quality of care received by patients. This includes the construction of several health facilities, the increase in the training of health workers, especially nurses, and the introduction of incentive packages (such as salary increase) to motivate health workers. The important question is to what extent does the institutional arrangement between the health facilities and the government as well as between health workers and public health facility administration affect the quality of care?

The objective of this study is to find the effect of institutional factors on the quality of care.

The institutional factors examined were mainly the extent of decentralization between government and health facilities, as well as between health workers and facility administration, the hiring procedure, and job satisfaction.

**Methods:**

The study used primary data on former patients from sixty six health facilities in three administrative regions of Ghana: the Northern, the Ashanti and the Greater Accra regions. The quality indicator used was effectiveness of treatment as determined by the patient. Ordered logit regression was run for the indicator with patient and health facility characteristics as well as institutional factors as independent variables. The sample size was 2248.

**Results:**

The results showed that the patient’s level of formal education had a strong influence on the effectiveness of treatment. In addition, effectiveness of treatment differed according to the administrative region in which the facility was located, and according to the extent of decentralization between health facility and government. The quality of instruments used for treatment, the working conditions for health workers, and job satisfaction had no effect on the effectiveness of treatment.

**Conclusion:**

Decentralization, the flow of information from government to health facilities and from health facility administrators to health workers are important in ensuring effectiveness. The study recommends further decentralization between health facilities as well as between health workers and administrators. In addition, the study recommends the involvement of health facilities in malaria programs to ensure the flow of information needed for effectiveness of treatment.

**Electronic supplementary material:**

The online version of this article (doi:10.1186/s12913-015-0802-7) contains supplementary material, which is available to authorized users.

## Background

Ghana, a developing economy with a population of 25.37 million is located in the tropics and hence its population is affected by tropical diseases such as malaria, guinea worm disease, and buruli ulcer [1,2]. Malaria is the dominant disease in the country, being responsible for about 33 percent of all outpatient attendants and about 49 percent of under five admissions [3]. Effective treatment of malaria is therefore important for the health of the Ghanaian population. As a result, there is high government involvement in the provision of treatment of the disease [3,4] and hence subject to institutions related to governance. The government constantly reviews existing methods of treatment and introduces new programs for prevention and treatment of the disease [3].

A strong health system that ensures reduction in morbidity and mortality from diseases requires the active networking of many stakeholders such as government, the health sector, and community members. When institutions function properly, productive networking could enhance the ability of the health sector to provide effective treatment of diseases. Because people seek treatment in order to reduce or eliminate morbidity, enhancing treatment effectiveness is welfare improving to patients and the population as a whole. It is therefore important to know the institutional factors that affect the effectiveness of malaria treatment in the Ghanaian economy.

Effectiveness of treatment refers to the impact of healthcare on the health status of the patient according to the patient’s evaluation [5]. When treatment is effective patients’ welfare improves. With public health facilities in the Ghanaian health sector providing care to a large percentage of the population [6], improvement in effectiveness of treatment in public health facilities is likely to be benefited by a large percentage of the population. The objective of this study is to examine the effect of institutional factors on the effectiveness of malaria treatment in the Ghanaian public health facilities. The focus of institutions is mainly on the extent to which the institutional arrangement between health facilities and the government as well as between health workers and public health facility administration affect the effectiveness of treatment.

### Institutional relationships in the Ghanaian health sector

Two main institutional relationships are of interest in the study. The first institutional relationship of interest is that between government and health facilities and this relationship is represented in the study as, the extent of decentralization in decision making, and the flow of information (on new treatment methods) from government to health facilities. The other institutional relationship of interest is that between health facility administration and health workers, and is represented in the study as the extent of decentralization, the flow of information (on new treatment methods) from health facility administration to health workers, opportunities for professional development of health workers, procurement process, and job satisfaction of health workers.

The definition of decentralization involves the transfer of responsibilities from a high level of government to lower levels [7]. The literature on decentralization however presents several definitions using various approaches such as the public administration approach, the local fiscal choice approach, social capital approach, and the principal agent approach [7]. The approach that is relevant to the current study is the principal-agent approach which focuses on the relationship between the government (the principal) and the health facility (agent), and how the principal can induce the desired behavior from the agent. Under this approach, decentralization involves the principal transferring responsibilities to the agent. Two levels of decentralization were of interest in this study: decentralization at the central government level, i.e., between government and health facilities (external decentralization), and decentralization within the health facility, i.e., between the facility administration and the health workers (internal decentralization).

As far as decentralization is concerned, public health facilities in the Ghanaian health sector can be divided into two: teaching hospitals and non-teaching hospitals. Teaching hospitals have autonomy in hiring health workers and can directly purchase drugs, equipment and other supplies and pay suppliers [8]. Teaching hospitals then enjoy external decentralization. The non-teaching health facilities however are governed by the Ghana Health Services (GHS) and cannot hire health workers directly; neither can they purchase other physical inputs without approval from the GHS at the various directorates [8].

External decentralization is expected to enhance the ability of the local health facilities to respond quickly to local needs [9]. It follows then that a teaching hospital in the Ghanaian health system can quickly restock when it runs out of drugs or hire new health workers without waiting for the government. Teaching hospitals are therefore more likely to respond quickly to the healthcare needs of patients to ensure effective treatment. It is therefore hypothesized that teaching hospitals provide more effective treatment than non teaching hospitals. Internal decentralization is needed to enhance treatment effectiveness. Given that health workers are directly in touch with patients, they are likely to have information on patients’ welfare issues. Involving health workers in decision making then is likely to enhance effectiveness of treatment. The study expects a positive relationship between effectiveness of treatment and internal decentralization.

Since methods of treatment change overtime [10] it is important that information on new methods is easily conveyed from government to the facilities. In addition, adequate flow of information from administrators to health workers also ensures that new methods of treatment are easily communicated to health workers to improve the effectiveness of treatment.

Treatment effectiveness is likely to be enhanced if the procurement process at the facility level ensures that quality equipment and inputs are supplied. Good working conditions for health workers could improve job satisfaction on the part of the workers and hence motivate them to improve the quality of treatment input to ensure effectiveness [11,12]. It is therefore hypothesized that the availability of these institutional factors enhances effectiveness of treatment.

Even though studies have examined the effect of decentralization on health outcomes, the focus of health outcome was on development of complication [13] and hence patients who did not develop complications but did not fully recover from their illness were not included in the analysis. The current study focused only on malaria outpatients because using the treatment of only one disease makes comparison less complicated, and in this way the effect of various factors on effectiveness need not be linked to the type of disease. Contrary to the literature that has focused on development of complications [e.g., 13], by using effectiveness of treatment in general for patients who had received treatment from selected health facilities, the current study was able to include those who did not develop complications but were not able to regain the health status before illness.

## Methods

### Data source and description

The survey data used for the study came from health facilities in three of Ghana’s ten administrative regions as outlined in the Appendix. The selected regions were the Greater Accra region, the Ashanti region, and the Northern region. The three regions were selected because the three public teaching hospitals in the country were located in those regions, with one hospital per region. The selected health facilities were three teaching hospitals, three regional hospitals (one from each region), twelve district hospitals (four from each region) and eight health centers (three from Ashanti, three from Greater Accra and two from the Northern region). Teaching hospitals were in general tertiary hospitals but they also had outpatient departments, and acted as the referral hospitals for the other hospitals. Administratively, Ghana is divided into ten regions and each region has a regional hospital that acts as the referral hospital for the region. Regions with teaching hospitals then had two referral hospitals with the teaching hospitals acting as referral to the regional hospitals as well as district hospitals. District hospitals acted as the referral hospitals to the health centers. The teaching and regional hospitals were located in the regional capitals and hence in large cities, while the district hospitals were spread over small towns and large cities.

The survey which was sponsored by the African Economic Research Consortium was done in 2010 and it involved interviewing patients, health workers and health facility administrators, with ethical approval from the Ghana Health Service on clearance ID: GHS-ERC:01/1/10. A sample of the questionnaires for the survey can be found in the Additional file 1. The authors obtained informed consent from respondents. The sampling frame for health workers was all nurses and doctors who have worked in the outpatient department within a month before the interview. In the case of patients, the sampling frame was all those who had received outpatient malaria treatment from the selected health facilities within a month to the interview. Each patient interviewed then had received outpatient malaria treatment in one of the selected facilities within a month before the interview. The patients were therefore former patients of the selected health facilities and were interviewed outside the premises of the health facilities. Information on patients’ contact in medical records was not adequate because not all patients had phone numbers or house addresses. Using medical records to select patients then could result in a weak representative sample of the population as only patients with addresses, who are also likely to be the affluent in society, would be interviewed. The study then did not use information on medical records to select patients. The researchers then approached anybody they met on the street or in any house that they entered and asked them if they had received outpatient malaria treatment from the nearest selected health facility in the community or town or city within a month. The respondents who were selected for interview then were those who responded positively to the question and were willing to be interviewed.

To select a house, the researchers first went to the streets in the residential areas in the community or areas served by each of the selected health facilities. For each street, the houses in each block were given numbers and the numbers were written on pieces of paper. The researchers then randomly picked any three of the numbers and entered those selected houses for interview. If respondents in the selected houses could not be interviewed, a number was picked from the remaining numbers to select another house. This continued for each facility until the desired sample size for the facility was reached. Note that the larger health facilities such as the district, regional and teaching hospitals serve wide areas beyond the neighborhood residents, and so interviewing people on the commercial streets near these facilities ensured that people who lived far but sought care from the selected facilities were also interviewed. The method of selection used then is likely to cover a good percentage of patients who have been treated by the facilities.

The choice of sample size was problematic because information on population of outpatients treated was not available for all selected health facilities. The selection of sample size of patients and health workers therefore depended on the type of health facility with the largest facilities getting the largest sample size of outpatients. In general, one hundred patients were selected in each teaching hospital, eighty from each regional hospital, and twenty from each health centre. The sample size for the district hospital patients ranged between eighty and sixty depending on the size of the facility. The choice of exact numbers is simply based on financial feasibility. Again, due to financial reasons, only fifty percent of health workers who worked in the outpatient department, nurses and doctors, were interviewed in each facility. The unit of observation was patients and the sample size reduced from 2890 to 2248, after removing observations with missing values. Hence 642 patients were dropped from the data.

### Sample

Outpatients were used in the study for two reasons. First, since malaria was a very common disease, the study wanted to find the extent to which the various facilities were capable of treating mild malaria which is the basic disease. Second, health facilities were included in the explanatory variables. The choice of health facility is correlated with severity of illness as sick people are likely to choose quality health facilities and severity of illness also affects effectiveness of treatment [14]. Since there was no variable for severity of illness and severity of illness is likely to be high among inpatients, the inclusion of inpatients could cause high correlation between health facility type and the error term. The use of outpatient is likely to ensure a uniform severity, mild malaria, and hence reduce any correlation between severity illness and the choice of health facility.

### Empirical specification

The empirical model used in the study is specified below:

1 $$ {y}_i={\beta}_1+{\beta}_2{X}_{2i}+{\beta}_3{X}_{3i}+{\beta}_4{X}_{4i}+{e}_i $$

where *yi* is malaria outpatients’ ranking of treatment effectiveness from selected health facilities. The variable is an ordinal variable with values between 1 and 7, with 1 representing poor effectiveness and 7 representing excellent effectiveness.

The *X*_*2i*_ is a vector of variables on patients’ characteristics including age, gender, education and occupation. Since health deteriorates with age [15], older patients were expected to have poorer outcomes than the young patients. Education is likely to enhance health as the educated individual is better equipped with information on health improvement than the less educated [15]. Effectiveness of treatment was therefore expected to improve with the level of education. Since there was no information on income (a weakness of the data), the type of employment of patients was used as a proxy for income. The employment categories used were unemployed (control group), those employed in the formal sector and those employed in the informal sector. Another dummy was added for farmers and non farmers (control group). People in the informal sector are often self employed and so bear a higher opportunity cost [16] of staying home when sick than the unemployed. Given that malaria treatment requires a lot of rest [17], people in the informal sector are less likely to rest when they have malaria and hence are more likely to report low effectiveness of treatment. Formal sector employees are often able to get sick days off work to rest and so are likely to comply with treatment and hence expected to report a higher effectiveness of treatment than the unemployed. Farmers belong to the informal category and the rationale for the inclusion of a dummy for farmers was the seasonal nature of their work. Their opportunity cost for rest, and hence compliance with treatment is likely to vary depending on whether or not it is within the planting and harvesting seasons. Since the survey was done in the planting season, farmers were therefore expected to report low effectiveness than non farmers.

The *X*_*3i*_ is a vector of health facility characteristics. These are dummy variables for teaching hospitals, and non teaching hospitals which are further dummied as health centers, district hospitals, and regional hospitals with health centers as the control group. As already explained the dummy for Teaching hospital was used as a proxy for external decentralization and so its coefficient was expected to be positive. Regional hospitals in Ghana have more autonomy in procurement than district hospitals, and a similar difference exists between district hospitals and health centers. The coefficients of regional and district hospitals were expected to be positive implying better effectiveness as a result of the ability to respond more quickly to medical needs compared with health centers.

Two additional dummies were also created for the administrative region in which the facility was located. Since the data were collected from three of the ten administrative regions in Ghana only two dummies were created. The three regions are the Greater Accra region, the Ashanti region, and the Northern region which was the control variable. These dummies are linked to governance because Ghana follows the central government system of governance and the capital city is located in the Greater Accra region. Facilities located in this region were expected to have the best in effectiveness of treatment as a result of their closeness to the head office of the Ministry of Health. The Ashanti region has the second largest city of the country and the largest population and so health facilities in this region are likely to receive more attention from the Ministry of Health in resource allocation than the Northern region which is the poorest of the three. Two more variables, number of doctors and nurses in each health facility, were also added making the number of variables in the vector of health facility characteristics seven. The coefficients of number of doctors and of nurses were expected to be positive as increase in personnel, structural quality, could improve effectiveness of treatment.

The third vector, *X*_*4i*_, has six dummy variables on the institutional factors. These factors are external flow of information, internal flow of information, quality of instruments used, inclusion of health workers in decision making (internal decentralization), opportunity for professional development, and job satisfaction. All these factors were expected to have a positive effect on the effectiveness of treatment. External flow of information refers to the flow of information between government and the health facility regarding new methods of treatment. The internal flow refers to information flow between facility administrators and health workers. A fast flow of such information was expected to enhance effectiveness of treatment. Similarly, quality instrument was expected to be positively correlated with effectiveness as it enhances diagnoses and treatment. Finally, professional development and job satisfaction, because they improve the skills and motivation of health workers, were also expected to have a positive impact on effectiveness of treatment. The last variable in the model is the error term.

The institutional variables were originally all coded between one and ten, with one implying poor ranking and ten representing excellent ranking. With the exception of ‘information flow from government to facility’ which was provided by health facility administrators, the information in the remaining institutional variables was provided by health workers. For the purposes of the estimation dummy variables were created for the institutional variables. To create the dummy variables, first the codes provided by health workers in each facility were averaged. A variable was created with the averaged values for each health facility. A dummy variable was thus coded as one when the averaged code was greater than 5. For example, the dummy variable for job satisfaction was coded as one whenever job satisfaction was on average rated by the health workers in a facility as greater than five. The implication is that the facilities in which health workers rated their job satisfaction on average above five were, on average, satisfied with their job.

Ordered logistic regression was used to estimate the model. The choice of ordered logit was due to the nature of the dependent variable which was made up of integers from 1 to 7 representing patients’ ranking of the effectiveness of treatment. The values in the variables were the observed ranking for the latent variable, y*, of the actual ranking. Letting y* = *xβ* + *ε* where y* is the exact unobserved ranking of effectiveness of treatment; *x* is a vector of independent variables described above [18]. When y* is equal to the lowest threshold, its observed value is 1 and the observed values increase until 7 for the highest threshold. With *yi* in (1) as y* and *xβ* as the coefficients and explanatory variables described above, the ordered logistic regression estimates the coefficients and the thresholds. A positive sign of the coefficient means that the latent variable increases with the regressor [18]. SPSS, version 16, was used to run the regression. The thresholds were not reported as they were not of importance in the discussion. For ease of interpretation, odds ratios were reported.

## Results

As shown in Table 1, the average age of patients was 36.6 years. More than half (59 percent) were women. The proportions of patients in the Greater Accra and the Ashanti regions were about the same while that of the Northern region was smaller. The respondents were mostly educated at the JSS and SSS levels. Consistent with the Ghanaian labor market, the patients were mostly informal sector workers [19]. As a result of the large number of district level facilities selected, patients who received care from district hospitals formed almost half of the sample followed by those in the health centers and then teaching hospitals. There were 38 public district level hospitals and 307 public health centers in the three selected regions. As shown in Table 1, these two types of health facilities provided treatment to most of the patients in the regions. Further analysis of the data showed that the distribution of female patients over the facility type followed that of the data and more than half of the patients in each hospital type were women. The proportion of women (62 percent) in the regional hospitals and health centers were slightly more than those in the teaching and district hospitals where women formed 51 percent and 54 percent respectively of the total patients.

**Table 1:** **Characteristics of study participants and facilities (n = 2248)**

**Variable**	**Total**	**Percentage**
Age		36.6 years (average)
Gender		
female	1,326	59
male	922	41
Greater Accra	897	39.9
Ashanti	861	38.3
Northern	526	21.7
Education		
• Uneducated (control group)	243	10.8
• Primary	360	16
• Junior Secondary School (JSS)	879	39.1
• Senior Secondary School (SSS)	470	20.9
• Tertiary	297	13.2
Occupation		
• Formal	450	20
• Informal	1214	54
• Farmer	225	10
• Unemployed	584	26
Teaching Hospital		
	330	14.7
Regional Hospital	119	5.3
District Hospital	1,086	48.3
Health Centre	713	31.7
Females in Hospital Types		
• Teaching Hospital	171	7.6
• Regional Hospital	74	3.3
• District Hospital	589	26.2
• Health Centre	447	19.9
Information flow from government to facility	407	18.1
Information flow from facility to health workers	1729	76.9
Quality of instruments	1,893	84.2
Internal decentralization	922	41
Opportunity for professional development	2,021	89.9
Job satisfaction	1,657	73.7

Table 1 shows that health workers in about 74 percent of the health facilities had job satisfaction. The administrators of 49 percent of the health facilities rated the flow of information from government to the facility as good or adequate. Again, health workers from about 77 percent of the health facilities on average, responded that there was adequate flow of information between the facility administration and the health workers. However, health workers from only 41 percent of the health facilities recorded decentralization at the facility level. More than 80 percent of the health facilities were rated by health workers as providing opportunity for professional development and following a procurement process that ensured the purchase of quality instruments.

Table 2 shows that most of the patients ranked the effectiveness of treatment above 4 implying that the health facilities were in general able to provide adequate treatment to malaria patients.

**Table 2:** **Patients’ view on effectiveness of treatment, with 1 = poor and 7 = excellent**

**Effectiveness of treatment**	**Total**	**Percent**
1	40	1.8
2	50	2.2
3	88	3.9
4	223	9.9
5	438	19.5
6	858	38.2
7	551	24.5

Table 3 reports the odds ratio from the regression. The results on patient characteristics show that effectiveness of malaria treatment improved with age and that effectiveness was not affected by the gender of the patient. The odds of a patient with tertiary education, having the highest ranking of effectiveness versus the remaining lower rankings was 1.52 times greater than for the uneducated, holding all other variables constant. Following a similar interpretation of the of the odds ratio, the results also show that patients who worked in the formal sector also reported higher effectiveness than the unemployed.

**Table 3:** **Patient characteristics, facility characteristics and institutional factors associated with treatment effectiveness**

**Independent variables**				
	**Odds ratios**	**P-value**	**95% confidence**	**Interval**
*X*_*2i*_ *– Patient characteristics*				
Age	1.01	.**024**	1.001	1.041
Gender (Female)	0.83	.470	0.720	1.211
Primary Education	0.89	.815	0.759	1.085
Junior Secondary School	1.02	.**005**	1.005	1.601
Senior Secondary School	1.46	**.018**	1.448	1.972
Tertiary Education	1.52	**.005**	1.518	2.090
Formal	1.55	**.004**	1.519	2.188
Informal	0.82	.084	0.684	1.017
Farmer	0.88	.375	0.734	1.129
*X*_*3i*_ *– Facility characteristics*				
Greater Accra Region	2.28	.**000**	2.072	2.671
Ashanti Region	0.99	.912	0.894	1.767
Teaching Hospital	6.33	.**000**	6.670	6.933
Regional Hospital	0.57	.**010**	0.503	0.914
District Hospital	0.98	.807	0.968	1.358
Number of nurses	0.99	.257	0.992	2.061
Number of doctors	0.99	.400	0.839	1.354
*X*_*4i*_ *– Institutional Factors*				
Quality of items procured	1.13	.481	1.078	1.857
Professional development	1.16	.371	1.153	2.061
Information flow to health workers	1.04	.**018**	1.007	1.567
Internal decentralization	1.39	.304	0.938	1.410
Information flow from government	2.37	.**004**	2.085	2.398
Job Satisfaction	0.87	.793	0.788	1.210

Variables with bold P-values are statistically significant at 5 percent significant level.

The results on the facility characteristics also show that, as expected, higher effectiveness of treatment was found in the Greater Accra region than the Northern region. The odds of a patient from the Greater Accra region choosing the highest ranking (7) versus any of the remaining lower rankings of treatment, was 2.28 times greater than patients from the Northern region holding all other variables constant. However, no statistically significant difference was found in the odds between the Ashanti and the Northern regions. Patients who received treatment in teaching hospitals found treatment more effective than those who received care in health centers. Specifically, the odds of a patient from teaching hospital choosing the highest ranking of effectiveness of treatment (7) versus any of the remaining lower rankings was 6.33 times greater than patients who received treatment in the health centers, holding all other variables constant. However those who received care in the regional hospitals found treatment less effective than those who received care in the health centers. For patients who received care from regional hospitals, the odds that they choose the highest ranking of effectiveness versus any of the lower rankings was 0.57 times lower than patients who were treated at health centers, holding all other variables constant. There was no statistically significant difference in effectiveness between health centers and district hospitals. Neither the number of nurses nor the number of doctors in the facility affected the effectiveness of treatment.

Only two of the institutional factors, the flow of information between government and health facilities, as well as that between health facility and health workers, were found to be statistically significant meaning that there was a relationship between effectiveness and the two institutional factors. The odds that a health facility with high flow of information between it and the government gets the highest ranking of effectiveness versus the lower ranking was 2.37 greater than a health facility with low flow of information, holding all other variables constants. Similarly, the odds that a health facility with high flow of information between the health facility administrator and health workers had the highest treatment ranking versus a lower ranking was 1.04 greater than a health facility with lower flow of information. In other words, effectiveness of treatment was ranked high in facilities with good flow of information from the government to the facility and from the facility administrators to health workers. The remaining four institutional variables were not statistically significant. Thus internal decentralization, quality of instruments used, professional development, and job satisfaction were not important in ensuring effectiveness of malaria treatment.

## Discussion

The present study demonstrates that older age, a higher education and employment within the formal sector has a positive correlation with treatment effectiveness. The results on hospital characteristics showed that greater effectiveness was reported in Greater Accra region and teaching hospitals than other two regions and health center respectively, while patients from regional hospitals reported lower reported effectiveness than health centre. Thus it is possible that patients from the Greater Accra region were advantaged by their location in the same region as the head office of the Ministry of Health. The results on institutional factors showed positive relationship between information flow, both externally (between government and health facility) and internally (between health facility administration and health workers), and effectiveness of treatment.

The study also showed that increasing the number of nurses in the health facility does not necessarily affect the health outcome of patients in the outpatients department. The inverse effect of number of doctors on effectiveness could imply that the large facilities do not allocate enough doctors to the outpatient department and hence perform poorly in effectiveness. These results are contrary to what was found in the literature. Needleman et al. [13] examined the relationship between structural and outcome quality. Specifically, the study sought to examine the effect of hospital staffing, focusing mainly on number of nursing care, on health outcomes. The paper showed that high proportion of hours of nursing care was associated with lower risk of patients having complications such as urinary tract infection. Stanton and Rutherford [20] also examined the effect of nurse-staffing levels on patient adverse outcomes such as pneumonia and urinary tract infection. The study showed a negative relationship between patient adverse outcomes and the level of nurse staffing. A similar result might have been obtained in the current study if the variable for the number of nurses referred only to the number of nurses in the outpatient department.

Given that the teaching hospital variable represents external decentralization, the results here imply that giving more autonomy to health facility in terms of hiring of health workers, and in procurement can improve the effectiveness of treatment provided. Internal decentralization however did not have any effect on treatment effectiveness. Studies have examined the effect of decentralization on health outcomes. For example Robalino, et al., [21] showed that fiscal decentralization in health facilities improved health outcomes in environments with high corruption but decentralization was less beneficial in environments with high ethno-linguistic fractionalization. Ethno-linguistic fractionalization simply refers to divisions according to ethnicity or language. When there is a high probability that any two people picked from a population belong to different ethnicities, health outcome is likely to be poor probably as a result of poor coordination [21]. The Ghanaian population is made up of seventy-five ethnic groups [22] and so it is possible that high ethno-linguistic fractionalization exists among health workers and administrators and hence impedes coordination. This could explain the weak impact of internal decentralization on treatment effectiveness.

Studies on the relationship between job satisfaction and health outcomes focused on the health outcome of health workers and not that of patients. For example, a study found that job dissatisfaction is associated with mental problems of health workers [23]. There is thus paucity of studies examining the effect of job satisfaction, as well as the institutional factors examined in the current study, on patient health outcome or effectiveness of treatment. The results in the current study imply that effective treatment of malaria can still be provided even if health workers were not satisfied with their job.

The results on institutional factors also imply that in order to provide effective treatment of malaria, the facility should be able to receive information from the government on new methods of treatment and any such information should be relayed to health workers. Such information flow is more capable of improving effectiveness of treatment than even the opportunity for professional development. The statistical insignificance of the procurement process that ensures the purchase of quality of instruments used implies that health facilities that rated their procured instruments to be of high quality did not provide better effectiveness of treatment than those who rated the quality low. This could be due to government regulation regarding the types of instruments imported or procured by the health facilities. If government’s policy ensures that instruments used met the set standard then variation in quality of instruments used may not statistically affect the effectiveness of treatment. A Pearson correlation between internal decentralization and the flow of information to health workers was 0.28 which is low. Thus, the insignificance of internal decentralization may not be due to high correlation with the flow of information to health workers. Internal decentralization then may be important for other aspects of quality of care such as attitude of health workers [24] but not the effectiveness of treatment.

### Limitations

Since information on population treated by health facilities was not available for the computation of an appropriate sample size the convenience sampling method. The usual disadvantage of convenience sampling is the possibility of using a sample size that is not a good representation of the population and hence cause biased results. The disadvantage in the current study is the possibility that the convenience sampling method used might not maintain the exact proportion in outpatient population across the health facilities. Such a disadvantage was minimized by ensuring that the sample size for larger facilities was always larger than those for the smaller facilities. A second limitation of the study is that data on number of nurses and doctors per facility was not limited to outpatient nurses and doctors but all doctors and nurses in the facilities. The results on these two variables then were interpreted with caution. The data lacked variables that measure patients’ income and length of training or working experience of doctors/nurses. Besides, the study did not compare the Ghanaian setting with the settings of other countries and hence the study is unable to generalize results to other settings.

Another limitation is that the results in the study were obtained from self reported data. The literature has raised concern over the validity of self reported morbidity. Subramanian et al. [25] for example, used Indian data to show that self reported illness was positively correlated with respondents’ socioeconomic status measured by the level of education. Thus respondents with high level of education were likely to report higher health status than the less educated. Such correlation is likely to bias the results on self reported health outcome so the current study validated the self reported effectiveness of illness by including patients’ characteristics including their level of education in the regression equation. In this way the study controls for any bias caused by personal characteristics. The study showed higher effectiveness of treatment among patients with higher education level such as Senior Secondary and Tertiary. This is consistent with Subramanian et al. [25].

## Conclusions

The study has shown that in addition to patients’ characteristics especially regarding education level, institutional factors are very important determinants of the effective treatment of malaria. The institutional factors examined were external decentralization, quality of instruments procured, opportunity of professional development, the flow of information between government and health facilities and between health facilities and health workers, and job satisfaction. Regional differences in effectiveness of treatment were also examined.

External decentralization, i.e., allowing health facilities some autonomy to hire and fire health workers and directly procure equipment has a positive effect on the effectiveness of treatment. The flow of information between health facilities and government as well as between health facility administrators and health workers were found to be also important determinants of the effectiveness of malaria treatment. Effectiveness of treatment was also found to be higher in the Greater Accra region than the Ashanti and Northern regions. Three recommendations are made for the improvement of the effectiveness of treatment.

There is the need for extensive study on the extent of external decentralization that could help improve the effectiveness of treatment in the non-teaching health facilities. Specifically, more studies are needed to examine how increased autonomy to health facilities in the hiring and firing of health workers as well as in the direct procurement of supplies needed for operations could improve the effectiveness of treatment. Secondly, national malaria programs should involve health facilities to ensure constant flow of information on new methods of treatment. In this way, facilities would be able to adopt new and effective methods of treatment at a faster rate and hence improve the effectiveness of treatment. Thirdly, policy adjustments are needed to ensure that other administrative regions have access to information of malaria treatment techniques as Greater Accra.

## Appendix

Source: http://cdn.ghanaweb.com/GHP/img/pics/59387424.gif.

## Additional file

Additional file 1:
**Questionnaire** **http://www.biomedcentral.com/imedia/1226524392108155/supp1.docx.**

## References

[1] The World Bank, http://www.worldbank.org/en/country/ghana

[2] World Health Organization, Fact Sheets: Tropical Diseases, (2013). http://www.who.int/topics/tropical_diseases/factsheets/en/

[3] National Malaria Control Programme: Annual Report 2009. Accra; 2009, http://ghanahealthservice.org/ghs-subcategory.php?cid=4&scid=41

[4] National Development Planning Commission: The Implementation of the Growth and Poverty Reduction Strategy (GPRS II) 2006–2009, 2006 progress report, http://siteresources.worldbank.org/INTPRS1/Resources/383606-1092340662634/cr09237.pdf

[5] Chalkley M, Malcomsom J, Culyer A, Newhouse J (2000). Government Purchasing of Health Service. Handbook of Health Economics. Volume 1A.

[6] Ghana Health Service, Health Sector in Ghana, Facts and Figures, 2010, http://www.moh-ghana.org/UploadFiles/Publications/GHS%20Facts%20and%20Figures%202010_22APR2012.pdf

[7] Couttolenc B: Decentralization and Governance in the Ghanaian Health Sector. Washington, DC: World Bank. https://openknowledge.worldbank.org/handle/10986/9376 License: CC BY 3.0 IGO, 2012

[8] Government of Ghana: Ghana Health Service and Teaching Service ACT 525, http://laws.ghanalegal.com/acts/id/140/ghana-health-service-and-teaching-hospitals-act

[9] Gross R, Bendassat J, Niral N, Cohen M (1992). Quality of care in decentralized primary care clinics: a conceptual framework. Int Journal of Health Planning & Mgt.

[10] WHO: Antimalarial Drug Efficacy for the Treatment of Uncomplicated Falciparum Malaria (2003). http://whqlibdoc.who.int/Hq/2003/WHO_HTM_RBM_2003.50.pdf

[11] Haas JS, Cook EF, Puopolo AL, Burstin HR, Cleary PD, Brennan TA (2000). Is the professional satisfaction of general internists associated with patient satisfaction?. J Gen Intern Med.

[12] Bakotić, D. Fiskovića, C, Relationship between Working Conditions and Job Satisfaction: The Case of Croatian Shipbuilding Company, International Journal of Business and Social Science, 4(2), 2013, http://ijbssnet.com/journals/Vol_4_No_2_February_2013/22.pdf

[13] Needleman J, Buerhaus P, Mattke S, Stewart M, Zelevinsky K (2002). Nurse-staffing levels and the quality of care in hospitals. N Engl J Med.

[14] Gowrisankaran G, Town RJ (1999). Estimating the quality of care in hospitals using instrumental variables. J Health Econ.

[15] Grossman M: The Human Capital Model. In Handbook of health economics. Volume 1A. Edited by Culyer A, Newhouse J; 2000

[16] Jain N: Improving Targeting of the Poor and Ensuring Equity: Emerging Systems and Approaches, Unpublished presentation by giz,http://www.nhis.gov.gh/files/Improving%20Targeting%20of%20the%20Poor%20and%20Ensuring%20Equity%20Ghana.pp

[17] Parasites in Humans, http://www.parasitesinhumans.org/

[18] Cameron A, Trivedi P (2005). Microeconometrics: methods and applications.

[19] Otoo KN, Osei-Boateng C, Asafo-Adjaye P: The Labour Market in Ghana: A descriptive analysis of the labour market component of the Ghana Living Standard Survey (V). http://www.ghanatuc.org/The-Labour-Market-in-Ghana.pdf

[20] Stanton M, Rutherford M: Hospital nurse staffing and quality of care. Agency for Healthcare Research and Quality, Research in Action Issue 14 No. 040029; 2004.

[21] Robalino D A, Picazo OF, Voetberg A: Does Fiscal Decentralization Improve Health Outcomes? Evidence from a Cross-Country Analysis http://dx.doi.org/10.1596/1813-9450%E2%80%932565

[22] Ghana Embassy: Population. www.ghanaembassy.org/index.php?page=population

[23] Faragher E, Cass M, Cooper C (2005). A relationship between job satisfaction and health, a meta analysis. Occup Environ Med.

[24] Amporfu E, Nonvignon J, Ampadu S (2013). Effect of institutional factors on the quality of care in the Ghanaian health sector. J Afr Dev.

[25] Subramanian SV, Subramanyam MA, Selvaraj S, Kawachi I (2009). Are self-reports of health and morbidities in developing countries misleading? evidence from India. Soc Sc Med.

