# Supplementary material for: The role of institutions on the effectiveness of malaria treatment in the Ghanaian health sector
Source: BMC Health Serv Res. 2015 Apr 19;15:169. doi: 10.1186/s12913-015-0802-7 (PMC4408601; doi:10.1186/s12913-015-0802-7)
Supplement: Additional file 1: — Questionnaire http://www.biomedcentral.com/imedia/1226524392108155/supp1.docx. [file 12913_2015_802_MOESM1_ESM.docx]

**Effect of Institutional Factors on Quality of Care**

**Funding Agency: African Economic Resort Consortium**

**Questionnaire for the Facility Administrator**

**Name of Facility**: ____________________________

1. Machinery used for treatment

How much was allocated to the hospital for the list above in each of the past three years: 2007, 2008, and 2009?

2007 2008 2009

How much was actually spent on these machinery?

1. Relevance of procurement

Is there any discrepancy in terms of relevance and quality, between the items requested and the items received from the procurement office?

Choose one:

High discrepancy no discrepancy

| 1 | 2 | 3 | 4 | 5 | 6 | 7 | 8 | 9 | 10 |
| --- | --- | --- | --- | --- | --- | --- | --- | --- | --- |

1. Hiring
2. How many health care workers are required for your facility?

Doctors:_________________ Nurses:______________ Others (specify) _______________

1. How many health care workers work in your facility?

Doctors:_________________ Nurses:______________ Others (specify) _______________

1. To what extent is any understaffing due to the hiring procedure?

No correlation High correlation

| 1 | 2 | 3 | 4 | 5 | 6 | 7 | 8 | 9 | 10 |
| --- | --- | --- | --- | --- | --- | --- | --- | --- | --- |

1. Flow of information between government and the health institution.

To what extent do policy changes favour the operation of your health institution?

No favor high favour

| 1 | 2 | 3 | 4 | 5 | 6 | 7 | 8 | 9 | 10 |
| --- | --- | --- | --- | --- | --- | --- | --- | --- | --- |

1. The flow of information between facility administration and health care workers

How much procurement information (e.g., price) is revealed to health care workers/department?

No flow of info complete flow of info

| 1 | 2 | 3 | 4 | 5 | 6 | 7 | 8 | 9 | 10 |
| --- | --- | --- | --- | --- | --- | --- | --- | --- | --- |

1. Overcrowding

What is the doctor patient ratio? 2007__________2008_____________2009____________

What is the nurse patient ratio? 2007___________2008_____________2009____________

**Effect of Institutional Factors on Quality of Care**

**Funding Agency: African Economic Resort Consortium**

**Questionnaire for health care workers**

1. Relevance of procurement.

Is there any discrepancy, in terms of relevance and quality, between items requested and items received from the procurement office?

1. Relevance

Choose one:

High discrepancy no discrepancy

| 1 | 2 | 3 | 4 | 5 | 6 | 7 | 8 | 9 | 10 |
| --- | --- | --- | --- | --- | --- | --- | --- | --- | --- |

1. quality

Choose one:

High discrepancy no discrepancy

| 1 | 2 | 3 | 4 | 5 | 6 | 7 | 8 | 9 | 10 |
| --- | --- | --- | --- | --- | --- | --- | --- | --- | --- |

1. How would you evaluate the general functional state of the equipment in the facility

Choose one:

Below standard above standard

| 1 | 2 | 3 | 4 | 5 | 6 | 7 | 8 | 9 | 10 |
| --- | --- | --- | --- | --- | --- | --- | --- | --- | --- |

1. Job Satisfaction

Is there an opportunity of learning on the job by getting the chance to work in different departments of the health facility?

Choose one:

No opportunity High opportunity

| 1 | 2 | 3 | 4 | 5 | 6 | 7 | 8 | 9 | 10 |
| --- | --- | --- | --- | --- | --- | --- | --- | --- | --- |

Is there any opportunity for professional development through furthering of formal education and attendance of seminars/workshops?

Choose one:

No opportunity high opportunity

| 1 | 2 | 3 | 4 | 5 | 6 | 7 | 8 | 9 | 10 |
| --- | --- | --- | --- | --- | --- | --- | --- | --- | --- |

In general, how would you rand the level of your job satisfaction?

Choose one:

No satisfaction high satisfaction

| 1 | 2 | 3 | 4 | 5 | 6 | 7 | 8 | 9 | 10 |
| --- | --- | --- | --- | --- | --- | --- | --- | --- | --- |

1. The flow of information between facility administration and health care workers

To what extent are health care workers involved in important decision making that affects the operation of the facility and/or procurement?

Choose one:

No involvement high involvement

| 1 | 2 | 3 | 4 | 5 | 6 | 7 | 8 | 9 | 10 |
| --- | --- | --- | --- | --- | --- | --- | --- | --- | --- |

**Effect of Institutional Factors on Quality of Care**

**Funding Agency: African Economic Resort Consortium**

**Questionnaire for patients**

**Name of Facility**: ___________________________

1. What is your age? ____________________________________________
2. Gender (check one): Female ____________ Male _________
3. What is your level of education? _________________________________
4. What is your occupation? ______________________________________
5. What is your address (name of town, village, or city) _____________________
6. How long did you wait to see the doctor? ______________________________
7. Did the doctor/nurse communicate the treatment procedure clearly to you?

Choose one:

Poor communication excellent communication

| 1 | 2 | 3 | 4 | 5 | 6 | 7 |
| --- | --- | --- | --- | --- | --- | --- |

1. Was the nurse/doctor’s interaction with you cordial?

Choose one:

Very unfriendly very cordial

| 1 | 2 | 3 | 4 | 5 | 6 | 7 |
| --- | --- | --- | --- | --- | --- | --- |

1. Do you think you receive the proper treatment for your illness?

Choose one:

Ineffective treatment effective treatment

| 1 | 2 | 3 | 4 | 5 | 6 | 7 |
| --- | --- | --- | --- | --- | --- | --- |

**Effect of Institutional Factors on Quality of Care**

**Funding Agency: African Economic Resort Consortium**

**Questionnaire for researcher**

1. Overcrowding at the outpatient department

Were patients standing while waiting for treatment due to lack of seat/space

Day 1 ___________________________

Day 2 ___________________________

Day 3 ­­­­­­­­­­­___________________________

1. Overcrowding at the wards

Were patients lying on the floor due to lack of bed?

Day 1 _____________________________

Day 2 _____________________________

Day 3 _____________________________
